# Statin use, HMGCR expression, and breast cancer survival – The Malmö Diet and Cancer Study

**DOI:** 10.1038/s41598-019-57323-9

**Published:** 2020-01-17

**Authors:** Olöf Bjarnadottir, Maria Feldt, Maria Inasu, Pär-Ola Bendahl, Karin Elebro, Siker Kimbung, Signe Borgquist

**Affiliations:** 1 Division of Oncology and Pathology, Department of Clinical Sciences, Lund University, Lund, Sweden; 2 Department of Oncology, Skåne University Hospital, Lund, Sweden; 3 Department of Oncology, Aarhus University Hospital, Aarhus, Denmark

**Keywords:** Breast cancer, Tumour biomarkers

## Abstract

Statins, commonly used to treat hypercholesterolemia, have also been proposed as anti-cancer agents. The identification of a predictive marker is essential. The 3-hydroxy-3-methylglutaryl-coenzyme-A reductase (HMGCR), which is inhibited by statins, might serve as such a marker. Thorough antibody validation was performed for four different HMGCR antibodies. Tumor expression of HMGCR (#AMAb90619, CL0260, Atlas Antibodies, Stockholm, Sweden) was evaluated in the Malmö Diet and Cancer Study breast cancer cohort. Statin use and cause of death data were retrieved from the Swedish Prescribed Drug Register and Swedish Death Registry, respectively. Breast cancer-specific mortality (BCM) according to statin use and HMGCR expression were analyzed using Cox regression models. Three-hundred-twelve of 910 breast cancer patients were prescribed statins; 74 patients before and 238 after their breast cancer diagnosis. HMGCR expression was assessable for 656 patients; 119 showed negative, 354 weak, and 184 moderate/strong expressions. HMGCR moderate/strong expression was associated with prognostically adverse tumor characteristics as higher histological grade, high Ki67, and ER negativity. HMGCR expression was not associated with BCM. Neither was statin use associated with BCM in our study. Among breast cancer patients on statins, no or weak HMGCR expression predicted favorable clinical outcome. These suggested associations need further testing in larger cohorts.

## Introduction

Statins are medications most commonly used by patients with cardiovascular diseases and hypercholesterolemia. Cholesterol is produced by the mevalonate pathway, a metabolic pathway that also produces precursors for steroid hormones and isoprenoids^1^. Statins exert competitive inhibition of the rate-limiting enzyme of the mevalonate pathway, 3-Hydroxy-3-methylglutaryl-coenzyme A reductase (HMGCR), an enzyme that has been found to be differentially expressed in breast cancer tumors^2^. In recent years, attention in cancer research has been drawn to the mevalonate pathway since statins have been found to exert pleiotropic intratumoral effects, suggesting a possible effectiveness in cancer. Examples of these effects are induction of apoptosis and inhibition of proliferation^3^, and immunomodulatory properties, through inhibition of Ras and Rho family GTPases^4–6^, and by decreasing the production of inflammatory cytokines and activating CD8+ T cells^7^. In breast cancer, statins have also demonstrated some anti-neoplastic properties in preclinical studies of breast cancer cells^8–11^. These findings are supported by epidemiological data showing protective associations between statin use and breast cancer recurrence and thus prognosis^12–14^. However, biomarkers for selection of patients that may benefit from statins are needed.

Previous studies have explored the correlation between tumor cell expression of HMGCR and breast cancer prognosis, as well as the association with statin treatment. One study found that high levels of HMGCR tumor expression in breast cancer was associated with favorable clinicopathological characteristics, such as smaller tumor size, low histological grade, estrogen receptor (ER) positivity, and low proliferation^2^. Data were confirmed in another study cohort demonstrating HMGCR as an independent prognostic marker, associated with an improved recurrence-free survival, particularly in ER-positive tumors^15^. In a study based on a prospective cohort, The Breast Cancer (BC) Blood study, HMGCR expression was associated with less aggressive tumor characteristics^16^. Another study correlated HMGCR expression as a predictor of response to tamoxifen^17^. These findings evaluating HMGCR protein expression have, however, been challenged by gene expression data, which have shown *HMGCR* expression to be inversely associated with breast cancer recurrence rates^18,19^.

The specificity of the HMGCR antibodies previously used might impact on these controversies, and herein we have applied a novel and extensively validated anti-HMGCR monoclonal antibody. Thus, this study aimed to explore and clarify the association between statin use, HMGCR expression based on a novel antibody, and breast cancer prognosis.

## Results

### Statin use, HMGCR expression, and patient- and tumor characteristics

**Figure 1:**
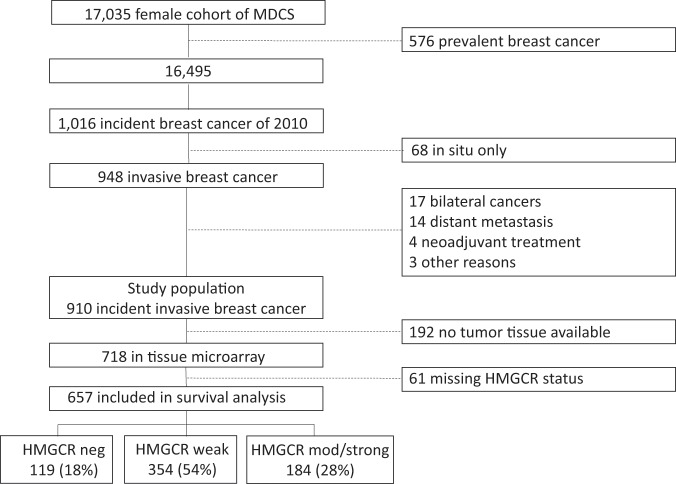
Flow chart showing the study population in the Malmö Diet and Cancer study. Dotted lines display reason for missing patients. Figure re-used from thesis of Olöf Bjarnadottir^20^.

By the end of follow-up time for identification of incident breast cancers by the 31^st^ of December 2010, a total of 1,016 breast cancers were diagnosed. After subtraction of patients diagnosed with cancer *in situ*, bilateral or distant metastatic breast cancer, 910 patients with invasive breast cancer were identified (Fig. 1 (figure as presented in the doctoral thesis of Olöf Bjarnadottir^20^). In 192 cases, tumor tissue was not available. The TMAs were thus constructed of biopsies from 718 patients, of which 61 were not possible to evaluate for HMGCR expression due to either inferior staining quality or lack of tumor tissue in the TMA core. In the end, 657 samples were available for annotation of HMGCR expression; 119 (18%) showed no expression, 354 (54%) weak expression, 169 (26%) moderate and 15 (2%) strong (Fig. 1 (figure as presented in doctoral thesis of Olöf Bjarnadottir^20^), Fig. S1).

A total of 312 patients from the study population of 910 had been prescribed statins during the years 2005 through 2014. Hereof, 74 patients were prescribed statins before (pre-diagnostic statin) and 238 women after (post-diagnostic statin) the breast cancer diagnosis, whereas 598 women had not been prescribed statin. Table 1 shows patient and tumor characteristics according to statin use. The distributions in the four groups (pre-diagnostic statin, post-diagnostic statin, any statin and never statin) were similar regarding body mass index (BMI) at baseline, tumor size, lymph node, and ER status. Proportionally, more patients in the pre-diagnostic statin group were diagnosed with grade III tumors, high Ki67, and higher HMGCR expression compared to the post-diagnostic and never statin groups. In comparison to patients never receiving statins, the patients in any statin group had higher BMI at baseline, and their tumors were more often PR positive (P < 0.01 and P = 0.01, respectively).

**Table 1:** Patient and tumor characteristics according to statin use.

All n = 910 Factor		Pre-diagnostic statin n ≤ 74 n (%) or mean (min–max)	Post-diagnostic statin n ≤ 238 n (%) or mean (min–max)	Any statin n ≤ 312 n (%) or mean (min–max)	Never statin n ≤ 598 n (%) or mean (min–max)	P-value any vs never statin use
**Age at baseline (n = 910)**	56.4 (44.7–73.0)	56.4 (46.8–71.5)	57.2 (44.9–72.8)	57.0 (44.9–72.8)	56.2 (44.7–73.0)	0.10^a)^
**Age at diagnosis (n = 910)**	65.5 (45.7–87.3)	71.1 (59.6–85.6)	64.9 (48.4–84.7)	66.3 (48.4–85.6)	65.0 (45.7–87.3)	0.02^a)∗^
**BMI at baseline (n = 910)**						
<25	466 (51)	29 (39)	98 (41)	127 (41)	339 (57)	
≥25 and <30	310 (34)	29 (39)	91 (38)	120 (38)	190 (32)	
≥30	134 (15)	16 (22)	49 (21)	65 (21)	69 (11)	<0.01^b)∗^
**Tumor size (n = 887)**						
≤20 mm	637 (72)	51 (72)	178 (76)	229 (75)	408 (70)	
>20 mm	250 (28)	20 (28)	57 (24)	77 (25)	173 (30)	0.15
**ALNI (n = 819)**						
Positive (≥1 metastatic node)	262 (32)	23 (33)	65 (30)	88 (31)	174 (33)	
Negative	557 (68)	47 (67)	151 (70)	198 (69)	359 (67)	0.58
**NHG (n = 835)**						
I	227 (27)	12 (17)	63 (28)	75 (26)	152 (28)	
II	392 (47)	32 (46)	112 (51)	144 (49)	248 (46)	
III	216 (26)	26 (37)	47 (21)	73 (25)	143 (26)	0.85^b)^
**ER status (n = 760)**						
Positive (>10%)	671 (88)	58 (87)	183 (89)	241 (89)	430 (88)	
Negative (≤10%)	89 (12)	9 (13)	22 (11)	31 (11)	58 (12)	0.84
**PR status (n = 689)**						
Positive (>10%)	378 (55)	49 (77)	106 (57)	155 (62)	223 (51)	
Negative (≤10%)	311 (45)	15 (23)	81 (43)	96 (38)	215 (49)	0.01^∗^
**HER2 status (n = 593)**						
Positive	52 (9)	6 (15)	14 (8)	20 (10)	32 (8)	
Negative	541 (91)	33 (85)	158 (92)	191 (90)	350 (92)	0.65
**Ki67 (n = 633)**						
Low (≤10%)	419 (66)	17 (40)	138 (73)	155 (67)	264 (66)	
High (>10%)	214 (34)	25 (60)	51 (27)	76 (33)	138 (34)	0.72
**HMGCR expression (n = 657)**						
negative	119 (18)	5 (8)	33 (18)	38 (16)	83 (19)	
weak	354 (54)	33 (52)	103 (58)	136 (56)	225 (52)	
moderate/strong	184 (28)	26 (41)	42 (24)	68 (28)	121 (28)	0.43^b)^

BMI: body mass index; ALNI: axillary lymph node involvement, NHG: Nottingham histological grade; ER: estrogen receptor, PR: progesterone receptor; HER2: Human epidermal growth factor 2; TNBC: triple negative breast cancer; HMGCR: HMG-CoA reductase; TAM: tamoxifen; AI: aromatase inhibitors. Pearson X2 test if not specified otherwise. ^a)^Linear regression. ^b)^Linear-by-Linear association. P-value between any statin treatment and never statin treatment. ^∗^P < 0.05. Pre-diagnostic statin use: statin prescribed before breast cancer diagnos. Post-diagnostic statin use: statin prescribed after breast cancer diagnos. Any statin use: patients prescribed statin at any point; either pre-diagnostic and/or post-diagnostic. Never statin use: patients never prescribed statin.

Table 2 shows the patient- and tumor characteristics according to HMGCR expression. The mean age at diagnosis was higher in patients with HMGCR moderate/strong tumors compared to the patients with HMGCR low or negative tumors. HMGCR moderate/strong tumors were associated with tumors with higher histological grade, high Ki67, and ER-negative tumors (P < 0.01, P < 0.01 and P < 0.01, respectively, Table 2).

**Table 2:** Distribution of patient and tumor characteristics of the study population, according to HMGCR expression.

All n = 910 Tumor in tissue microarray, n (%)		Yes, 718 (79)					No, 192 (21)
HMG-CoA reductase (HMGCR) expression assessable, n (%)		Yes, 657 (92)				No, 61 (8)	
HMGCR expression; negative, weak, moderate/strong		HMGCR negative ≤ 119 (18)	HMGCR weak ≤ 354 (54)	HMGCR moderate/strong ≤ 183 (28)	P-value		
**Factor n (%) or mean (min**–**max)**							
**Age at baseline, years (n** = **910)**	56.4 (44.7–73.0)	56.1 (44.7–72.4)	55.8 (44.9–73.0)	57.1 (46.0–73.0)	0.18^a)^	55.1 (45.7–72.7)	57.7 (44.8–72.8)
**Age at diagnosis, years (n** = **910)**	65.5 (45.7–87.3)	64.1 (48.5–81.3)	65.1 (45.7–84.7)	67.3 (48.6–87.3)	<0.01^a)∗^	63.1 (49.4–84.4)	66.0 (47.8–85.1)
**BMI at baseline (n** = **910)**							
<25	466 (51)	72 (60)	163 (46)	98 (53)		36 (59)	97 (50)
≥25 and <30	310 (34)	33 (28)	124 (35)	64 (35)		18 (29)	71 (37)
≥30	134 (15)	14 (12)	67 (19)	22 (12)	0.71	7 (12)	24 (13)
**Tumor size (n = 887)**							
≤20 mm	637 (72)	77 (65)	243 (69)	129 (70)		49 (82)	139 (80)
>20 mm	250 (28)	41 (35)	109 (31)	55 (30)	0.40	11 (18)	34 (20)
**ALNI (n = 819)**							
Positive (≥1 metastatic node)	262 (32)	42 (36)	124 (37)	57 (32)		7 (14)	32 (23)
Negative	557 (68)	74 (64)	207 (63)	124 (68)	0.32	45 (86)	107 (77)
**NHG (n = 835)**							
I	227 (27)	29 (25)	101 (29)	27 (15)		23 (42)	47 (35)
II	392 (47)	72 (61)	170 (49)	69 (38)		20 (36)	61 (46)
III	216 (26)	16 (14)	76 (22)	86 (47)	<0.01^∗^	12 (22)	26 (19)
**ER status (n = 760)**							
Positive (>10%)	671 (88)	101 (92)	297 (91)	137 (79)		35 (85)	101 (93)
Negative (≤10%)	89 (12)	9 (8)	30 (9)	36 (21)	<0.01^∗^	6 (15)	8 (7)
**PR status (n = 689)**							
Positive (>10%)	378 (55)	60 (60)	165 (56)	84 (53)		17 (47)	52 (53)
Negative (≤10%)	311 (45)	40 (40)	131 (44)	75 (47)	0.26	19 (53)	46 (47)
**HER2 status (n = 593)**							
Positive	52 (9)	2 (2)	22 (9)	21 (15)		2 (5)	5 (6)
Negative	541 (91)	85 (98)	224 (91)	119 (85)	<0.01^∗^	36 (95)	77 (94)
**Ki67 (n = 633)**							
Low (≤10%)	419 (66)	80 (83)	190 (71)	68 (47)		20 (56)	61 (69)
High (>10%)	214 (34)	16 (17)	79 (29)	76 (53)	<0.01^∗^	16 (44)	27 (31)
**Statin use (n = 910)**							
Pre-diagnostic statin use	74 (8)	5 (4)	33 (9)	26 (14)		1 (2)	9 (5)
Post-diagnostic statin use	238 (26)	33 (28)	101 (29)	39 (21)		18 (29)	47 (24)
Any statin use	312 (34)	38 (32)	134 (38)	65 (35)		19 (31)	56 (29)
Never statin use	598 (66)	81 (68)	220 (62)	119 (65)	0.67^★^	42 (69)	136 (71)

BMI: body mass index; ALNI: axillary lymph node involvement, NHG: Nottingham histological grade; ER: estrogen receptor, PR: progesterone receptor; HER2: Human epidermal growth factor 2; TNBC: triple negative breast cancer; HMGCR: HMG-CoA reductase; Pre-diagnostic statin use: statin prescribed before breast cancer diagnos. Post-diagnostic statin use: statin prescribed after breast cancer diagnos. Any statin use: patients prescribed statin at any point; either pre-diagnostic and/or post-diagnostic. Never statin use: patients never prescribed statin according to drug register. Linear-by-linear association if not specified otherwise. ^a)^Test of zero slope in a linear regression model. ^∗^P-value < 0.05. ^★^P-value between any vs never statin use.

### HMGCR antibody validation

**Figure 2:**
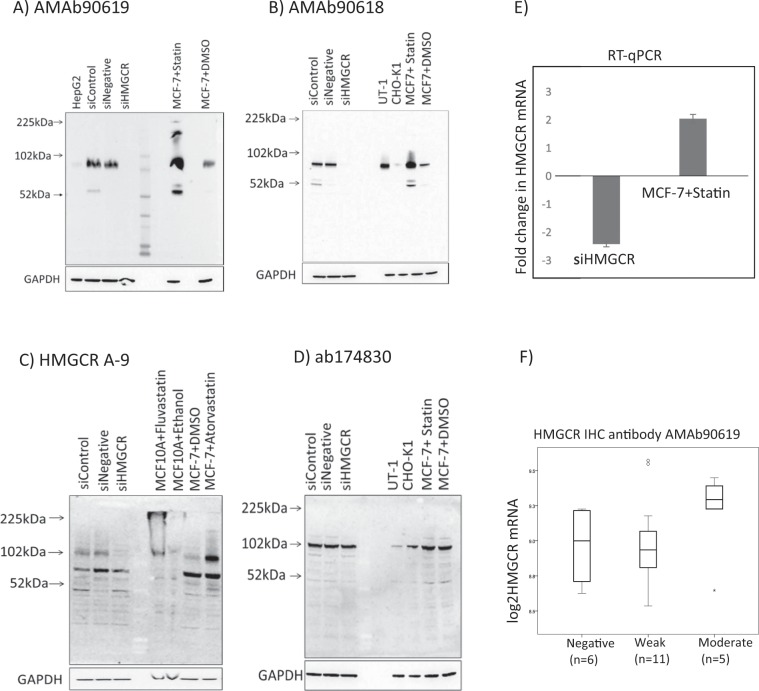
Validation of anti-HMGCR antibodies. (**A–D**) Western blots showing the expression of HMGCR after respective treatments tracked by different antibodies. All western images are full-length blots. (**A**) HMGCR AMAb90619, (**B**) HMGCR AMAb90618, (**C**) HMGCR A-9 and (**D**) HMGCR ab174830. Human breast cancer MCF-7 cell line was the main test cell line, while the HEPG2 liver cancer cell line and the Chinese hamster ovary cell lines CHO-K1 and UT-1 (derived from CHO-K1 following prolonged exposure to mevastatin) served as additional controls. (**E**) RT-qPCR was performed to evaluate the efficiency of downregulating *HMGCR* with siRNA or upregulating *HMGCR* with statin treatment. (**F**) Correlation of gene- and protein expression measured by antibody AMAb90619 in tumors. Figure re-used from thesis of Olöf Bjarnadottir^20^.

siRNA interference and statin treatment were used to downregulate and upregulate HMGCR expression, respectively, in MCF-7 cells. As illustrated in Fig. 2 (figure as presented in doctoral thesis of Olöf Bjarnadottir^20^), *HMGCR* mRNA levels were significantly reduced by about 2.41-folds relative to the controls following siRNA transfection (Fig. 2E). Likewise, statin treatment significantly upregulated *HMGCR* mRNA expression by about 2.05-folds relative to the controls (Fig. 2E). In Western blotting, all antibodies tested detected a protein band at about 100-kDa, which is the expected molecular weight of HMGCR (Fig. 2A–D). However, the differential modulation of HMGCR expression by siRNA or statin treatment was accurately tracked by all antibodies except HMGCR ab174830, which did not show any differential expression between siRNA silenced cells or statin-treated cells and controls (Fig. 2D). These data suggest that the HMGCR ab174830 antibody might be recognizing a different protein of similar molecular weight than HMGCR. AMAb90619, AMAb90618, and HMGCR A-9 reliably captured the differential effects of HMGCR knock-down and upregulation and showed a positive reaction in the additional positive control cell lines, confirming their specificity to the target protein. These antibodies also showed reactivity with a protein of approximately 55 kDa, especially after statin exposure, the identity of which is being investigated.

**Figure 3:**
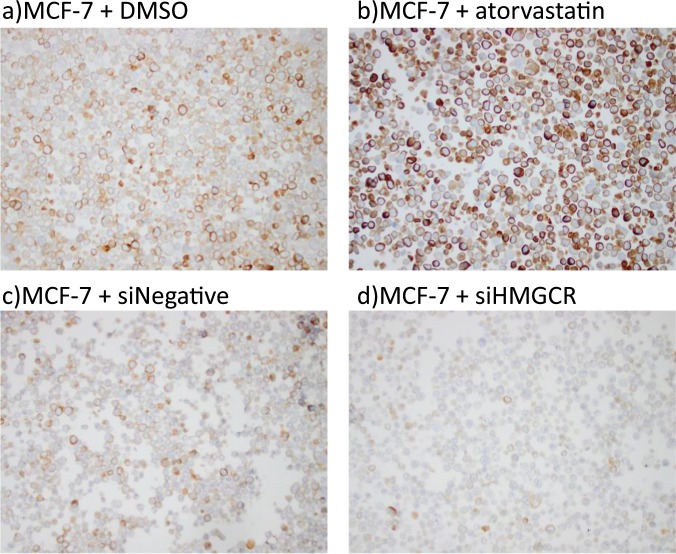
Immunocytochemistry images of HMGCR stainings on paraffin embedded MCF-7 cell pellets: (**a**) MCF-7 cells treated with vehicle control- DMSO. (**b**) atorvastatin treated MCF-7 cells. (**c**) MCF-7 cells treated with scramble siRNA (siNegative). (**d**) MCF-7 cells treated with HMGCR siRNA (siHMGCR).

Further, AMAb90619 was selected to extend the validation for the intended immunohistochemical analysis. We stained paraffin embedded MCF-7 cell pellets using immunocytochemistry and similar to the immunoblotting analysis the immunocytochemistry also showed a decreased intensity for the siRNA knockdown and a clear upregulation in the atorvastatin treated cells compared to the respective controls (Fig. 3). This antibody (AMAb90619) was also tested on a TMA containing a small collection of breast cancer tissue and cell lines, including liver tissue to serve as positive control for the staining. The expression of HMGCR was heterogeneous in the breast cancer cell lines and tissues, and a positive reactivity was seen in the liver, as expected. Therefore, this antibody was used for IHC analyses of HMGCR expression on the TMA, including all incident breast cancers from the MDCS, as reported herein.

### Breast cancer mortality by statin use

Analyses of associations between statin use and breast-cancer-related deaths were restricted to patients diagnosed with breast cancer from January 1^st^ 2006 onwards. There was no evidence to support an association between statin use and BCM in the age-adjusted analysis (HR_age-adj._ 0.73; 95% CI 0.35–1.52, P = 0.40, Table 3). Similar results were seen in the multivariate models adjusted for tumor characteristics and adjuvant treatment (Table 3). In the exploratory analyses stratified for HMGCR expression, statin use was not associated with BCM in patients with negative/weak HMGCR expression (HR_age-adj._ 0.37, 95% CI 0.11–1.24, P = 0.11, Table 3) nor in patients with moderate/strong HMGCR expression (HR_age-adj._ 0.77, 95% CI 0.19–3.10, P = 0.71, Table 3). Stratified analyses for ER status did not demonstrate any difference in protective effects of statin.

**Table 3:** Statins effect on breast-cancer specific mortality for breast-cancer patients diagnosed after 1st of January 2006.

	Crude					Model 1					Model 2					Model 3				
	HR	95% CI	P	Number patients	Breast cancer deaths	HR	95% CI	P	Number patients	Breast cancer deaths	HR	95% CI	P	Number patients	Breast cancer deaths	HR	95% CI	P	Number patients	Breast cancer deaths
Never statin use		ref					ref					ref					ref			
**All cases**																				
Statin use	0.78	0.38–1.62	0.51	293	37	0.73	0.35–1.52	0.40	293	37	0.69	0.31–1.52	0.36	255	31	0.80	0.34–1.91	0.62	223	28
**HMGCR negative/weak**																				
Statin use	0.67	0.21–2.10	0.49	148	15	0.37	0.11–1.24	0.11	148	15	0.30	0.07–1.26	0.10	137	13	0.32	0.06–1.61	0.17	117	12
**HMGCR moderate/strong**																				
Statin use	0.75	0.19–3.04	0.69	83	9	0.77	0.19–3.10	0.71	83	9	0.85	0.20–3.73	0.83	80	8	0.56	0.08–4.06	0.56	70	7

All cases and in subgroups for HMGCR expression.

Crude: unadjusted analysis; Model 1: adjusted for age; Model 2: adjusted for age, tumor size, tumor grade, lymph node involvement, ER status; Model 3: Model 2 with the addition of adjustment for planned adjuvant treatment (chemo-, endocrine- and radiation treatment). HMGCR: HMG-CoA reductase; HR: Hazard ratio; CI: Confidence interval.

### Breast cancer mortality by HMGCR expression

The possible prognostic role of HMGCR expression in breast cancer was evaluated for the entire study population with valid HMGCR expression (n = 657) and showed no evidence of associations (Table 4). No statistically significance was seen in the survival analysis when comparing patients with HMGCR negative tumors with patients expressing HMGCR moderate/strongly (Table 4). When restricting the analyses to ER-positive breast cancer only, there was no statistically significant difference (HR_age-adj._ 1.71, 95% CI 0.89–3.28, P = 0.11).

**Table 4:** Breast cancer mortality according to HMGCR expression in crude and adjusted models.

All patients	Total (n)	Events (n)	Crude			Model 1			Model 2			Model 3		
	657	110	HR	95% CI	P	HR	95% CI	P	HR	95% CI	P	HR	95% CI	P
HMGCR negative	119	20		ref	0.33		ref	0.33		ref	0.45		ref	0.56
HMGCR weak	354	52	1.04	0.62–1.74	0.90	1.04	0.62–1.74	0.90	1.21	0.70–2.10	0.49	1.17	0.67–2.06	0.58
HMGCR moderate/strong	184	38	1.39	0.80–2.39	0.24	1.39	0.80–2.39	0.24	1.47	0.80–2.68	0.21	1.41	0.75–2.63	0.29

Crude: unadjusted analysis; Model 1: adjusted for age; Model 2: adjusted for age, tumor size, tumor grade, lymph node involvement and ER status. Model 3: Model 2 and planned adjuvant treatment (chemo-, endocrine- and radiation treatment). HMGCR: HMG-CoA reductase; HR: Hazard ratio; CI: Confidence interval.

## Discussion

In this population-based prospective cohort study, we have investigated BCM according to statin use and studied HMGCR as a prognostic factor. Breast cancer patients prescribed a statin showed no evidence of reduced BCM compared to never users. There was, however, a trend towards lower BCM among statin using patients with no or weak HMGCR expression compared to patients with moderate or strong expression of HMGCR. Irrespective of statin use, HMGCR expression was significantly associated with more aggressive tumor characteristics, although no significant associations were observed for breast cancer-related mortality.

The associations between statin use and cancer-related mortality have been studied for some years, and the results have not been undisputed. Nielsen *et al*. have shown reduced cancer-related mortality among statin users, who were prescribed a statin before their cancer diagnosis^13^. In a study from Finland, both pre-diagnostic and post-diagnostic statin use was associated with lowered risk of breast cancer death^14^. Cardwell *et al*. found some evidence for reduced breast-cancer and all-cause mortality among post-diagnostic statin users^21^, while Emilsson *et al*. and Smith *et al*. found no association^22,23^. In a Danish study by Ahern *et al*. using breast cancer recurrence as the clinical endpoint, breast cancer patients taking simvastatin had a significantly improved breast-cancer free survival with 10 fewer breast cancer recurrences per 100 women after 10 years of follow-up^24^. In this study, we did not have access to breast cancer recurrence rates and were unable to confirm that statin use reduced BCM. Insufficient power in this cohort might be responsible for this lack of confirmation since reliable data on statin use was only valid from July 1^st^, 2005, when the Swedish Prescribed Drug Register was initiated.

In our study we did not find association between HMGCR expression, statin use and BCM. In patients treated with statins a lower BCM was seen in the tumor group that expressed HMGCR negatve/weak compared to tumors with moderate/high HMGCR expression, but this was not statistically significant. In our subanalysis with ER-positive respective negative status, we detected no difference in BCM. Furthermore, we could not see that HMGCR expression was more prognostically important for ER-positive patients and that statin use could be more preventive for ER-positive patients, as has been reported in a recent publication^25^. There, the prognostic impact of cholesterol-lowering medication (CLM) use in combination with endocrine treatment in ER-positive patients was studied and showed improved disease-free survival and distant recurrence-free interval for breast cancer patients that initiated CLM use during endocrine treatment^25^.

The rate-limiting enzyme of the mevalonate pathway, HMGCR, has been studied as a possible predictive marker for patients that would benefit from statin treatment in a cancer setting. We have earlier published the results from a window-of-opportunity breast cancer trial, where a decrease in tumor proliferation was seen only in tumors expressing HMGCR^26^. In previous studies evaluating the role of HMGCR in breast cancer, HMGCR expression has consistently been associated with prognostically beneficial tumor characteristics; i.e., low histological grade, expression of estrogen- and progesterone receptors, less axillary lymph node involvement^2,15,16^. In this study, however, patients with moderate/strong expression of HMGCR more often had tumors with grade III, ER-negative and high Ki67 compared to no or weak HMGCR expressing tumors. The use of a different antibody and breast cancer heterogeneity can partly account for these differences. In this study, a novel monoclonal antibody recently developed by Atlas Antibodies (https://atlasantibodies.com) was used to evaluate HMGCR expression. An ideal antibody for this purpose should be reproducible, perform well in the correct setting, and be specific for the target protein^27^. Previously we have used polyclonal antibodies from different vendors^2,15,26^, but in this study, we aimed to identify the most appropriate HMGCR antibody by performing in-depth validation of four different antibodies. The antibody chosen for this study was one of three antibodies that showed specificity to the desired HMGCR protein by capturing the differential effects of HMGCR knock-down and up-regulation, and also, showed a positive reaction in the positive control cell lines.

The prognostic impact of HMGCR in breast cancer has previously been evaluated by IHC in two independent cohorts; (1) a consecutive breast cancer cohort where patients with tumors expressing HMGCR had a significantly prolonged recurrence-free survival, also when adjusted for established prognostic factors^15^; and (2) a population-based cohort of primary breast cancer patients in Sweden, among whom HMGCR expression was not associated with disease-free survival^16^. In this study, we did not detect a statistical association between HMGCR expression and BCM.

Our study has some limitations. As previously mentioned, the Swedish Prescribed Drug Register started on the 1^st^ of July 2005, almost 15 years later than the first breast cancer case in 1991. In our analysis, we also gave the statin use the extra 6-month marginal time for plausible treatment effect by only selecting breast cancer cases from the 1^st^ of January 2006. Due to this, when evaluating statins effect on BCM, the breast cancer cases diagnosed up until the 31^st^ of December 2005 were not included in the analyses, resulting in fewer cases for the survival analyses. Additionally, we do not know how and if the patients actually took the statin they were prescribed. One study on statin use in an elderly population showed that after 6 months almost half of the patients stopped taking their tablets^28^. Although our study population was younger, we can probably assume that medication adherence was lower than prescription rates. Lastly, clinical outcome was restricted to breast cancer-related mortality as data on disease recurrence was unavailable, which has been the preferred outcome in previous studies.

To conclude, in this study we have investigated the association of statin use and HMGCR expression on BCM. We did not find association between HMGCR expression and BCM. Neither did we find evidence to suggest that statin use was associated with BCM. We observed associations between high HMGCR expression and unfavorable tumor characteristics, such as high tumor grade and high Ki67, although no independent association with BCM we detected. This should be further investigated in a larger observational study.

## Materials and Methods

### The Malmö Diet and Cancer Study (MDCS)

To examine associations between lifestyle factors and cancer, a population-based prospective cohort study, the Malmö Diet and Cancer Study, was initiated in Malmö, Sweden and enrolled healthy volunteers between 1991 and 1996^29^. All residents of Malmö by 1^st^ of January 1991 and born between 1926 and 1945 were invited to participate. Approximately 40% of the source population participated in the study^30^, and from the female population, a total of 17,305 women joined the study. At baseline, data were collected from interviews, questionnaires, and health examinations. Also, measures of body constitution and blood samples were taken. Written informed consent was obtained from all participants. Ethical permission was obtained from the Ethical Committee at Lund University (Dnr 472/2007). The study was performed in accordance with relevant guidelines and regulations.

### Study population characteristics and follow-up

Through linkage of the MDCS female cohort with the Swedish Cancer Registry and the South Swedish Tumor Registry, a total of 1,016 women were identified with an incident breast cancer diagnosed during follow-up until December 31^st^, 2010. This study only includes primary breast cancer cases and, thus, women with primary incident breast cancer diagnosed before enrollment in the MDCS (n = 576) were excluded. Information regarding vital status and cause of death was retrieved from the linkage of the MDCS breast cancer database to the Swedish Cause of Death Registry with the end of follow-up by December 31^st^, 2016.

### Statin use

With the Anatomical Therapeutic Chemical (ATC) Classification, information on statin use was obtained from the Swedish Prescribed Drug Register from July 2005, when the registry was initiated, through 2016.

### Tumor and histopathological analyses

Information on tumor histological type, size, Nottingham grade, and axillary lymph node involvement (ALNI) was retrieved from pathology reports for tumors diagnosed from 2005 and onwards, whereas immunohistochemical (IHC) assessed markers were obtained from tissue microarrays (TMAs) from 1991 to 2007^2^, and from pathological reports from 2008 and onwards for estrogen receptor (ER), progesterone receptor (PR), proliferation index (Ki67), and human-epidermal growth factor-2 (HER2) status. Breast cancers diagnosed before 2006 were re-evaluated regarding invasiveness, histological type, and grade^31^.

### HMGCR antibody validation

The specificity of several anti-HMGCR antibodies to the HMGCR antigen was validated by genetic and pharmacological strategies that alter the expression of HMGCR. The breast cancer cell line we used for antibody-validation was MCF-7, which is known to up-regulate HMGCR expression upon statin treatment. To upregulate HMGCR expression, MCF-7 cells were exposed to 5 µM atorvastatin (Sigma-Aldrich) for 24 h after which total protein and total RNA was extracted for Western blotting and RT-qPCR, respectively. Alternatively, to decrease the expression of HMGCR, the MCF-7 cells was transfected with 25 nM of ON-TARGET plus HMGCR siRNA pool (L-009811-00-0005, Human HMGCR 3156 siRNA, Dharmacon) constituted in DharmaFECT-transfection reagent (Dharmacon). Following 24 h of transfection, cells were maintained in full growth medium for another 48 h, after which total protein and total RNA were extracted for Western blotting and RT-qPCR respectively, as previously described^19^. Furthermore, HMGCR protein expression was assessed in the HEPG2 liver cancer cell line, Chinese hamster ovary cell lines CHO-K1 and UT-1 (derived from CHO-K1 following prolonged exposure to mevastatin) and fluvastatin treated MCF10A cell lysate (Kindly donated by Dr Linda Penn, Princess Margaret Cancer Centre, Toronto, Ontario, Canada) as additional positive controls. The following anti-HMGCR antibodies were screened; HMGCR AMAb90619 and HMGCR AMAb90618 (mouse monoclonals, 1:1000, Atlas Antibodies, Sweden), HMGCR ab174830 (rabbit monoclonal, 1:300, Abcam, Sweden), and HMGCR A-9 (mouse monoclonal, 1:500, kindly donated by Dr Linda Penn, Princess Margaret Cancer Centre, Toronto, Ontario, Canada). GAPDH expression served as a loading control for Western blotting and RT-qPCR experiments. To further validate the HMGCR antibody for immunohistochemical analyses, we performed immunocytochemistry on MCF-7 cells in which the HMGCR expression was altered as mentioned before. Cell pellets of MCF-7 cells were fixed in 4% formalin overnight, stained with hematoxylin, dehydrated and paraffin embedded. The paraffin embedded cell pellets were stained with HMGCR AMAb90619 antibody (1:75) using an automatic stainer (Autostainer plus, Agilent, DK).

### HMGCR expression

The HMGCR annotation with immunohistochemistry (IHC) was performed on TMAs that were constructed with duplicate 1-mm cores from each tumor (Beecher, WI, USA). Sections of 4 µm were cut from the TMAs. Before immunostaining, the sections were baked at 50 °C overnight and de-paraffinized in xylene and graded ethanol. Antigen retrieval was then performed using citrate buffer pH6 (ThermoFisher Scientific, Waltham, MA, USA) in a decloaking chamber (Biocare Medical, Walnut Creek, CA, USA). Sections were stained with the mouse monoclonal antibody against HMGCR (AMAb90619, CL0260, Atlas Antibodies, Stockholm, Sweden) diluted 1:100 in Autostainer 480S (ThermoFisher Scientific, Waltham, MA, USA) using a commercial kit (UltraVision LP HRP polymer®, Primary Antibody Enhancer, Ultra V Block and DAB plus substrate system®, ThermoFisher Scientific, Waltham, MA, USA). Slides were counterstained with hematoxylin and mounted using Pertex. Images of the stained slides were taken using an automated system (VSlide, Metasystems).

The web-based digital pathological platform PathXL Xplore (http://www.pathxl.com, PathXL Ltd., UK) was used for microscopy evaluations. The expression of HMGCR was evaluated based on cytoplasmic intensity using a four-grade scale; negative, weak, moderate, and strong. After the annotation, the strongly stained group of tumors was merged with the moderately stained group due to the low number of patients with strongly stained HMGCR (n = 15), resulting in three groups (negative, weak and moderate/strong). The HMGCR annotation was performed independently by two observers (OB and MF), both blinded to clinical and pathological data. The study obeys to the REMARK guidelines^32^.

### Statistical analysis

Associations between patient and tumor characteristics with statin use were evaluated and presented both as numbers and percentages. Continuous variables were summarized by mean, minimum, and maximum values. Distributional differences between the two groups “any statin use” and “never statin use” were assessed with X^2^ test or linear regression (X^2^ test for trend) as appropriate. The same methods were used to evaluate associations between patient/tumor characteristics and HMGCR expression.

The association between statin use, HMGCR expression, and prognosis was evaluated using breast-cancer-specific mortality (BCM) as a clinical endpoint. BCM was defined as the incidence of breast cancer-related deaths, both when breast cancer was considered the direct cause or the contributing cause of death. Follow-up time was calculated from the time of breast cancer diagnosis to the date of one of the following events; date of breast cancer-related death, date of death from another cause, date of emigration or the end of follow-up as of December 31^st^, 2016.

The associations between HMGCR expression and time to breast cancer-related death was analyzed by cause-specific Cox regression, yielding hazard ratios (HR) with 95% confidence intervals. The follow-up time was censored at the date of death from a cause not related to breast cancer – a so-called competing event. HRs should therefore, be interpreted in a hypothetical world where all other causes of death have been eliminated^33^. In addition to crude analyses, three multivariate models were fitted. Model 1 was adjusted for age at diagnosis (continuous). Model 2 was adjusted for age at diagnosis and tumor characteristics [tumor size > 20 mm (yes/no), metastatic lymph nodes (yes/no), histological grade (grade 1, 2, and 3), and ER status (positive/negative)]. Model 3 included the covariates of model 2 with the addition of planned adjuvant treatments (endocrine treatment (yes/no), chemotherapy (yes/no), and radiotherapy (yes/no)).

The prognostic impact of statin use was evaluated for patients diagnosed with breast cancer from 2006 and onwards since the statin data were retrieved from the Swedish Prescribed Drug Register, which was initiated in July 2005. For the evaluation of the relationship between statin and BCM, the same strategy for crude and adjusted analyses were done as described above. In an exploratory analysis, the predictive value of HMGCR regarding the association between statin use, BCM was evaluated through analyses stratified by HMGCR expression (HMGCR negative/weak and HMGCR moderate/strong, respectably). Statistical analyses were performed in SPSS 24.0 (IBM) and Stata version 14.1 (StataCorp LP, College Station, TX, USA).

### Ethical Standards

Ethical permission was obtained from the Ethical Committee at Lund University (Dnr 472/2007). Informed consent was obtained from all individual participants included in the study. The study was performed in accordance with relevant guidelines and regulations.

## Supplementary information

Supplementary Figure S1.
