# Supplementary material for: Statin use, HMGCR expression, and breast cancer survival – The Malmö Diet and Cancer Study
Source: Sci Rep. 2020 Jan 17;10:558. doi: 10.1038/s41598-019-57323-9 (PMC6969108; doi:10.1038/s41598-019-57323-9)
Supplement: Supplementary file 1 — Supplementary Figure S1. [file 41598_2019_57323_MOESM1_ESM.pdf]

Statin use, HMGCR expression, and breast cancer survival – The Malmö Diet and Cancer Study

Authors: Olöf Bjarnadottir, Maria Feldt, Maria Inasu, Pär-Ola Bendahl, Karin Elebro, Siker Kimbung, Signe Borgquist

Additional file 1: Figure S1

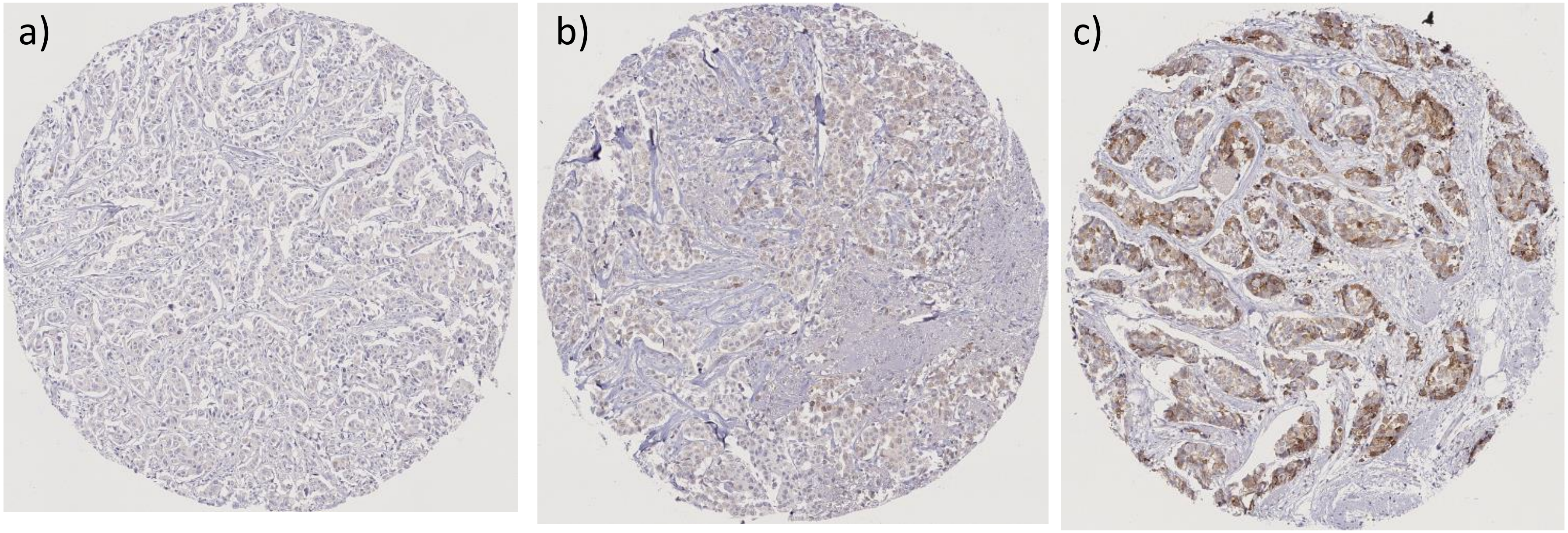

Supplementary Figure 1. Examples of immunohistochemical HMGCR staining with negative expression (a), weak expression (b), moderate/strong expression (c), respectively.
